# Efficacy of Transarterial Chemoembolization (TACE) for Early-Stage Hepatocellular Carcinoma

**DOI:** 10.3390/medicina59122174

**Published:** 2023-12-14

**Authors:** Moonhyung Lee, Hyun Phil Shin

**Affiliations:** Department of Gastroenterology and Hepatology, Kyung Hee University Hospital at Gangdong, Kyung Hee University College of Medicine, Seoul 05278, Republic of Korea; drmoon_0101@naver.com

**Keywords:** treatment outcome, transarterial chemoembolization, hepatocellular carcinoma, early stage

## Abstract

*Backgound and Objectives:* The treatments of choice for patients with early-stage hepatocellular carcinoma (HCC) are surgical resection, local ablation therapy, and liver transplantation; however, transarterial chemoembolization (TACE) is commonly performed due to variations among patients and liver diseases. This study aimed to assess the efficacy of TACE in patients with early-stage HCC. *Materials and Methods:* A retrospective analysis was performed of all TACE procedures performed at Kyung Hee University Hospital at Gangdong over a 15-year period (July 2006 to November 2021). The study included a total of 97 eligible patients with early-stage HCC ≤ 5 cm initially treated with TACE. The mean participant age was 63.47 ± 11.02 years; 69 were men (71.1%). The number of Child–Pugh class A patients was the highest (74 patients [76.3%]), followed by Child–Pugh class B (19 patients [19.6%]) and Child–Pugh class C (4 patients [4.12%]). *Results*: A complete response was achieved in 84 (86.6%) patients after the first TACE procedure, with 1-, 2-, and 3-year survival rates of 91.8%, 87.3%, and 75.4%, respectively. In the multivariate analysis, the patients with a low initial alpha-fetoprotein (AFP) ≤ 20 ng/mL (*p* = 0.02) and a complete response after the first TACE (*p* = 0.03) were associated with favorable overall survival. *Conclusions:* TACE can be used to treat patients with early-stage HCC who are unsuitable for ablation or surgery. If patients are well selected, TACE may be an alternative treatment for patients with low AFP levels who respond well to the initial TACE procedure.

## 1. Introduction

In Korea, hepatocellular carcinoma (HCC) is the sixth most common cancer [1]. Globally, HCC is the fifth most common cancer and the second most common cause of cancer-related deaths [2]. The global landscape of HCC is further nuanced by geographical variations, with a predominant concentration of cases in Asia. Several well-established risk factors contribute to the development of HCC, encompassing viral elements (such as chronic hepatitis B and C), metabolic influences (including diabetes and non-alcoholic fatty liver disease or NAFLD), toxic exposures (such as alcohol), and disorders related to the immune system. On a global scale, the implementation of successful Hepatitis B Virus (HBV) vaccination programs has led to a significant decline in HCC incidence among the mid-age population (30–59 years) [3].

Despite these strides, the persistent rise in HCC incidence and mortality signals a new challenge, primarily fueled by the obesity pandemic contributing to the prevalence of NAFLD. Projections indicate a worrisome trajectory, with a predicted 41% increase in global HCC mortality by the year 2040 [4]. This underscores the pressing need for comprehensive strategies to address the evolving landscape of HCC risk factors and incidence patterns.

In Korea, patients with HCC have experienced an increasing 5-year survival rate, reaching 35.6% in recent years, compared to 14.1% in the late 1990s and 28.2% in 2010. However, it is still classified as a cancer with a poor prognosis and a survival rate of <40% [5]. Although a unified staging system for HCC is lacking, the American Joint Committee on Cancer (AJCC) and Barcelona Clinic Liver Cancer (BCLC) staging systems are commonly used. The standard classification of cancer in oncology is based on the Tumor Node Metastasis (TNM) staging system. The eighth TNM edition of the AJCC for HCC has several limitations, including non-liver function or patient health status [6], while the BCLC staging system consists of three additional factors—tumor stage, liver function status, and performance status—and has been validated in several studies [7].

Surgical resection and local therapy are recommended radical treatment methods for early-stage HCC [8]. Surgical resection is a highly effective treatment for HCC with well-preserved liver function (Model for End-stage Liver Disease [MELD] score < 10 for compensated Child–Pugh class A liver function), and the tumor is localized on one side [9,10]. Local ablation is performed as first-line therapy in patients with a solitary HCC tumor < 2 cm, three or fewer tumors, and a tumor diameter < 3 cm [10]. There has been an increasing trend in the use of local therapy as an alternative to surgical resection, given its improved experience and outcomes [11]. Local therapy methods include radiofrequency ablation (RFA), microwave ablation, and laser ablation, which generate localized heat to treat tumors, and cryotherapy, which uses extremely cold temperatures for treatment [12].

In addition to HCC stage and liver function status, various factors, such as comorbidities and doctor preference, are considered in treatment decisions. According to the BCLC system, the recommendation for applying TACE gains prominence when other suggested treatments become unviable or ineffective during the early stages of HCC. This underscores the adaptive and versatile nature of TACE as a valuable intervention in the dynamic landscape of HCC management. Particularly noteworthy is the broad acceptance and recommendation of TACE in various clinical scenarios within Asian countries, reflecting the diverse applicability and success of this treatment modality in different contexts [13]. In Korea, transarterial chemoembolization (TACE) is the most common primary treatment [14]. While the specific clinical indications for TACE may exhibit slight variations across diverse staging systems, it stands as a cornerstone in the therapeutic arsenal for intermediate-stage HCC. The multifaceted nature of TACE, endorsed by its utilization across different clinical situations and geographic regions, accentuates its role as a well-established and adaptable strategy in the comprehensive approach to managing HCC.

Since 2004, the landscape of TACE has been shaped by two distinct techniques: conventional TACE (cTACE) and TACE with drug-eluting beads (DEB-TACE) [15]. This approach involves the strategic delivery of chemotherapy through transcatheter means, utilizing a lipiodol-based emulsion in conjunction with an embolizing agent. The synergy of these components produces robust cytotoxic and ischemic effects, establishing cTACE as an early and evidence-supported intervention. In a parallel development, the introduction of drug-eluting beads (DEBs) brought a new dimension to the TACE methodology. DEBs are designed to gradually release chemotherapeutic agents, elevating the intensity and duration of ischemic effects. This innovative approach reflects a nuanced evolution in TACE, emphasizing precision and controlled release for enhanced therapeutic impact.

Despite challenges posed by impaired liver function and concurrent comorbidities, surgical resection is limited in treating even early-stage liver cancer. Furthermore, local ablation may be hindered by difficult access or a propensity for bleeding. Consequently, this study seeks to assess the effectiveness of TACE as a primary therapeutic approach for early HCC, based on AJCC staging. The objective is to scrutinize treatment outcomes and identify factors predictive of a favorable prognosis following TACE administration.

## 2. Patients and Methods

### 2.1. Study Setting and Inclusion/Exclusion Criteria

The patients selected for this study were ≥18 years of age and diagnosed with early-stage HCC (Stage 1A or 1B based on the 8th edition of the AJCC staging framework) [16] between July 2006 and February 2023. TACE is the first treatment option for HCC. These patients were deemed unsuitable for surgery or thermal ablation by the local HCC multidisciplinary meeting (MDM) and underwent their initial transarterial chemoembolization (TACE) during the specified period, having had no prior intra-arterial treatment. Surgery ineligibility was determined by factors such as significant portal hypertension, substantial comorbidities, or ineligibility for liver transplantation. Similarly, ineligibility for standard thermal ablation was based on the risky or inaccessible anatomical location of the lesions, as evaluated by the attending radiologist at the MDM. Examples of risky locations included lesions near major portal/biliary branches, the bowel, diaphragm, gall bladder, subcapsular exophytic lesions, or percutaneously inaccessible lesions (hepatic dome). Also, the patient’s concerns about undergoing surgery, the physician’s assessment of a patient’s general condition, and the center’s preferences are considered.

Patients who met the following criteria were excluded:

1. Less than 18 years of age;
2. Died of a cause unrelated to HCC;
3. Lost to follow-up within 1 year after TACE;
4. Received alternative treatments after undergoing TACE.

### 2.2. TACE Protocols

Prior to TACE, the mesenteric and hepatic arteries were subjected to digital subtraction angiography. This diagnostic process aims to map the intricate vascular structure of the liver, evaluate the presence of arteriovenous connections, and identify the specific arteries supplying the tumor. Patients received local anesthesia and antiemetic medication before the procedure. TACE was performed by selectively catheterizing the segmental hepatic arteries supplying the lesion. A combination of lipiodol and doxorubicin was administered, followed by selective gel foam embolization. Lesion quantity and size determined the doses of lipiodol and doxorubicin administered during each procedure. The technical success of the TACE procedure was confirmed when the targeted lesions were completely embolized, and no technical complications were observed during treatment [17]. Only conventional TACE was performed in the treatment.

### 2.3. Follow-Up

All patients underwent regular haematological tests to evaluate periprocedural complications and their potential effects on liver function. Triphasic computed tomography (CT) or dynamic magnetic resonance imaging (MRI) was performed in all patients 1 month after TACE and at 3-month intervals thereafter. Furthermore, patient examinations were conducted on the same 3-month schedule. The tumor’s response to treatment was assessed on CT or MRI by expert abdominal radiologists using the amended Response Evaluation Criteria in Solid Tumors framework [18]. In this evaluation, a complete response (CR) was defined as the absence of intratumoral arterial enhancement in all designated target lesions. A partial response (PR) was defined as a reduction of at least 30% in the combined diameters of the viable portion of the target lesions using the initial sum of the diameters of the target lesions as a reference. Progressive disease (PD) was defined as an increase of 20% or more in the combined diameters of the viable target lesions, with the lowest sum of the diameters of viable (enhancing) target lesions recorded since the initiation of treatment as reference. Stable disease (SD) was defined as any case that did not meet the complete definitions of CR, PR, or PD.

### 2.4. Study Endpoint

The analysis of the therapeutic effect of TACE performed here was based on whether liver necrosis occurred at least 1 month after TACE. The patients in this group were observed and treated continuously, and the survival rate and survival period were analyzed after the initial treatment. The relative hazards of survival rates after TACE were analyzed based on factors such as liver function, tumor size, tumor markers, cirrhosis complications, and response to the first TACE procedure.

### 2.5. Statistical Analysis

The Cox proportional hazards model was used to analyze the data. On marginal screening, variables with a significance level of ≤0.2 were extracted. The extracted variables were subjected to multiple Cox regression analyses. Categorical variables are described as number (*n*) and percentage (%), while continuous variables are shown as mean ± standard deviation. The data were analyzed using RStudio (version 4.2.2; RStudio, Inc., Boston, MA, USA), and guidance from a statistical expert was sought for the data interpretation.

## 3. Results

### 3.1. Patient Characteristics

Table 1 shows the patients’ baseline characteristics and response to the first TACE. Ninety-seven eligible patients were included in this study. The mean participant age was 63.47 ± 11.02 years; 69 were men (71.1%). Most patients had Child–Pugh class A disease (74 patients [76.3%]), followed by Child–Pugh class B (19 patients [19.6]) and Child–Pugh class C (4 patients [4.12%]). A complete remission (CR) was achieved in 84 patients (86.6%) after the first TACE procedure. There were no occurrences of serious complications aside from post-embolization syndrome after TACE. In the univariate analysis, a low MELD score (*p* = 0.00), a low initial AFP≤ 20 ng/mL (*p* = 0.02), and a normal range of prothrombin time (INR) (*p* = 0.00) were associated with favorable overall survival (OS).

### 3.2. Survival Graph

**Figure 1:**
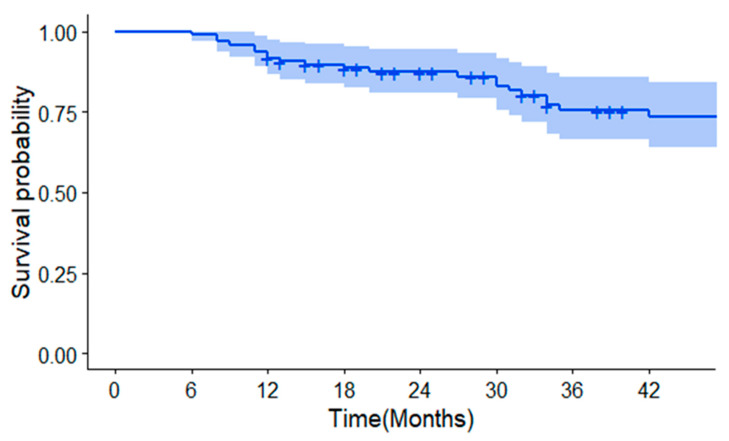
Kaplan–Meier analysis of survival probability.

Figure 1 shows the Kaplan–Meier analysis of the survival probability of patients after TACE. The 1-, 2-, and 3-year survival probabilities were 91.8%, 87.3%, and 75.4%, respectively.

**Figure 2:**
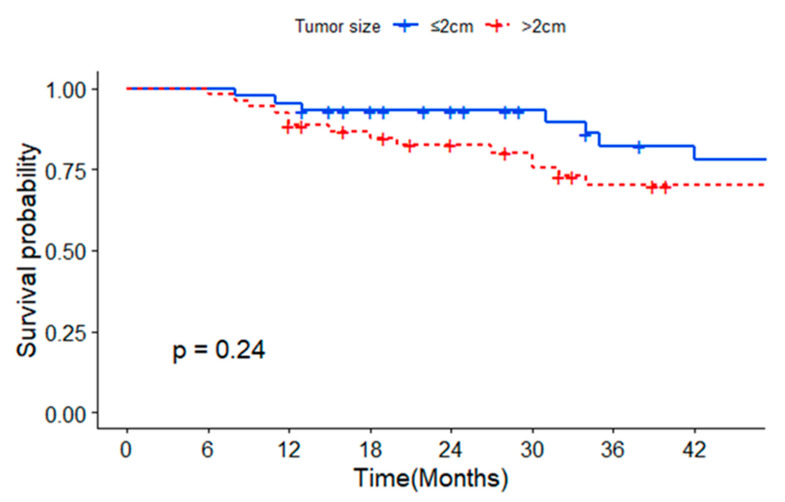
Survival probability by tumor size.

Figure 2 shows the Kaplan–Meier analysis of survival probability by tumor size. The 1-, 2-, and 3-year OS rates of patients with tumors < 2 cm were 89%, 86%, and 70%, respectively, whereas those of patients with tumors > 2 cm but <5 cm were 80%, 72%, and 57%, respectively. Tumor size did not significantly affect survival (*p* = 0.24).

**Figure 3:**
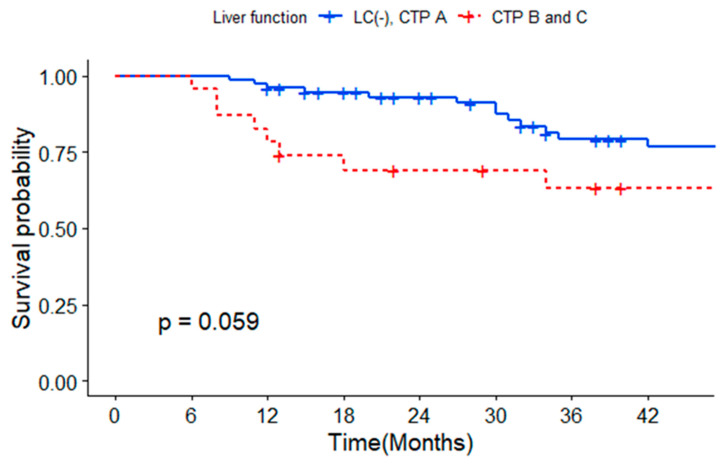
Survival probability according to presence of cirrhosis and Child–Pugh class.

Figure 3 shows the Kaplan–Meier analysis of survival probability by liver function. The OS rates of patients without cirrhosis or cirrhotic patients with Child–Pugh class A disease at 1, 2, and 3 years were 91%, 87%, and 90%, respectively, while those of patients with cirrhosis and Child–Pugh class B or C disease at 1, 2, and 3 years were 78%, 69%, and 63%, respectively.

### 3.3. Hazard Ratio

**Figure 4:**
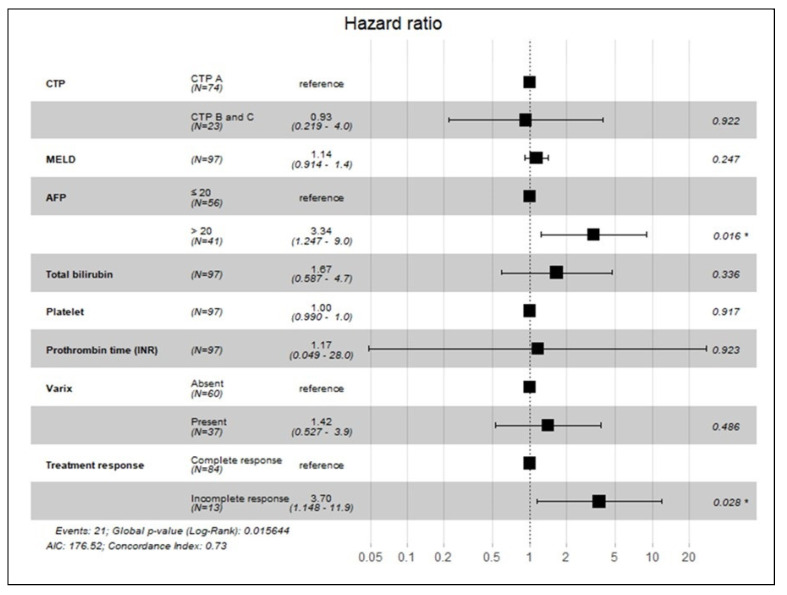
Subgroup analysis of mortality among patients treated with transarterial chemoembolization. Statistically significant factors with *p* < 0.05 (*).

In the multivariate analysis (Figure 4), the risk for mortality was statistically significant for an initial AFP > 20 ng/mL (*p* = 0.02) and an incomplete response after the first TACE (*p* = 0.03).

## 4. Discussion

In the present context of South Korea, men and women aged > 40 years who are at a high risk of developing liver cancer (cirrhosis or hepatitis B or C carriers) underwent abdominal ultrasound and AFP testing every 6 months as part of the national cancer screening program. Consequently, the probability of detecting early-stage HCC has increased significantly. TACE is generally considered the first-line treatment for unresectable intermediate-stage HCC in patients with adequate liver function. Randomized controlled trials have demonstrated improved survival of patients with unresectable tumors after TACE compared with the best supportive care [19]. Nevertheless, there is a lack of evidence supporting the use of TACE as an initial therapeutic approach for patients diagnosed with very early- or early-stage HCC (AJCC 1A, 1B).

Hashem et al. conducted a retrospective review on using drug-eluting beads (DEB-TACE) to managevery early- or early-stage hepatocellular carcinoma (VES-HCC) [20]. In the study (BCLC 0 and A), the complete remission rate was 45.9%, and the median survival was 71.1 months. In our study, early-stage HCC patients, including those with Child–Pugh class B and C disease, were analyzed for their survival rates. The CR rate was 86.4%, and the OS rates of patients with Child–Pugh class B or C disease at 3 years were 63%. Certainly, it is not a direct head-to-head comparison, but this analysis offers valuable insights into the variations in the effectiveness of TACE across diverse BCLC stages.

In addition, Yang et al. reported the most effective initial treatment strategy for small hepatocellular carcinoma (SHCC) through Bayesian network meta-analyses [21]. In conclusion, TACE was highly efficacious (58.9%) at decreasing the rates of major complications. The fact that there were no significant complications observed, even with the inclusion of patients with CP-B and C in our study, suggests that TACE demonstrated a notable advantage in terms of safety during the procedure.

This study demonstrated promising outcomes after TACE as the primary treatment for early-stage HCC, even in patients with Child–Pugh class B and C status. In the subgroup analysis, the OS rates of patients with tumors < 2 cm at 1, 2, and 3 years were 89%, 86%, and 70%, respectively, whereas those of patients with tumors > 2 cm but < 5 cm at 1, 2, and 3 years were 80%, 72%, and 57%, respectively. These findings do not suggest an inferiority of TACE to RFA or surgical intervention [15,22]. In the univariate analysis, a low MELD score (*p* = 0.00), a low AFP ≤ 20 ng/mL (*p* = 0.02), and a normal range of prothrombin time (INR) (*p* = 0.00) were significantly associated with favorable OS. In the multivariate analysis, an initial AFP ≤ 20 ng/mL (*p* = 0.02) and a CR after the first TACE (*p* = 0.03) were associated with OS.

TACE is generally recognized as safe, with the most common complications being elevated liver enzyme levels and fever. Serious complications include liver abscess, liver infarction, and cholecystitis [23,24]. In this study, despite the inclusion of patients with Child–Pugh class B and C disease, no major complications occurred. Liver function test results were mild and transitory, represented by an increase in alanine transaminase and bilirubin levels, which returned to baseline values within a few days.

Despite these promising outcomes, TACE remains a conventional treatment method due to concerns about recurrence. The recurrence rate of HCC was 35.8% in patients with tumors > 3 cm that did not respond to TACE versus 1.9% in patients with tumors ≤ 3 cm and a CR to TACE [25].

While our study exclusively employed conventional TACE, there are many technologically advanced forms beyond it. An alternative endovascular treatment known as drug-eluting bead transarterial chemoembolization (DEB-TACE) uses microspheres to release chemotherapeutic agents within a target lesion, ensuring controlled pharmacokinetics [26]. In Dr. Zhang’s study [27], loading HepaSphere microspheres with a combination of hypertonic saline and non-ionic contrast material effectively reduces their size. Employing this method to load epirubicin (ndTACE) enhances drug concentration in tumors, resulting in an improved antitumor effect. Therefore, besides conventional TACE, various modified embolization methods can be employed to improve treatment outcomes. Kim et al. investigated preoperative balloon-occluded transcatheter arterial chemoembolization (B-TACE) in 25 solitary hepatocellular carcinoma (HCC) patients [28]. Successful B-TACE was followed by 8% major and 24% minor complications. CT revealed a 96% complete response, and pathological analysis showed 72% complete HCC necrosis. Therefore, B-TACE proves safe and effective, acting as a valuable bridge treatment for solitary HCC.

Hepatocellular carcinoma (HCC) presents a highly diverse immune environment, influencing the response to various embolization methods and subsequently impacting the efficacy of immunotherapy [29]. Despite the continued prominence of locoregional treatments (LRTs) in HCC therapies, about half of patients eventually resort to lifelong systemic treatments. Procedures such as TACE, SIRT, and thermal ablation significantly enhance the immunosuppressive state of HCC. However, this condition can be mitigated by combining these treatments with immunotherapy, aiming to restore lymphocyte activity and cellular immune factor secretion. The integration of immune treatment with both locoregional and systemic approaches has brought about a profound transformation in HCC management. It is evident that combining locoregional treatments with immunotherapy could yield distinctive therapeutic outcomes compared to conventional methods.

The primary therapeutic approaches for early and very early-stage hepatocellular carcinoma include surgical methods like liver transplantation or surgical resection (SR), ablation techniques such as radiofrequency ablation (RFA), microwave ablation (MWA), cryotherapy ablation (CRA), percutaneous ethanol injection (PEI), no-touch radiofrequency ablation, non-catheter-based treatments like stereotactic body radiotherapy (SBRT), and catheter-based embolic interventions such as transarterial chemoembolization (TACE). The European Association for the Study of the Liver (EASL) [10] and the American Association for the Study of Liver Diseases (AASLD) [30] advocate for surgical resection as the primary treatment for early and very early-stage hepatocellular carcinoma.

However, for patients ineligible for surgery, ablation emerges as a viable and effective alternative. Ablation achieves the necrosis of neoplastic cells by altering the local temperature, offering several advantages such as minimal invasiveness, high safety, cost-effectiveness, and reproducibility. Among ablative techniques, radiofrequency ablation (RFA) stands out as the most prevalent and effective alternative therapy to surgery in selected patients [31]. SBRT is also a rising local treatment modality, demonstrating robust local control rates of 91% for tumors smaller than 5 cm and 74% for tumors measuring 5 cm or larger [32]. Conventional TACE exhibited a recurrence rate in cases of early-stage HCC with a complete response to treatment (CR) that was not higher than observed in surgery as well [20,33].

In addition to conventional interventional methods, immunotherapy has emerged with heightened promise, particularly when synergistically combined with adjuvants such as curative resection, surgical ablation, or TACE [34]. Combinatorial immuno-therapeutic approaches, exemplified by the pairing of atezolizumab and bevacizumab, showcase encouraging outcomes. This success has paved the way for recommendations advocating their administration not only in advanced stages but also in early-stage interventions, marking a paradigm shift in the landscape of treatment strategies for improved patient outcomes. Hence, the optimal treatment options for very early- or early-stage HCC remain controversial, with no consensus reached. Considering these encouraging findings in our study regarding early-stage HCC, there is a pressing need to reevaluate the role of TACE as a legitimate modality for controlling disease progression in specific cases.

This study has several strengths. This study included patients with hepatic function impairment classified as Child B and C with ascites and esophageal varix. It might be anticipated that patients with severe hepatic function impairment could have a higher likelihood of complications following TACE. However, in this study, no major complications, apart from post-embolization syndrome, were observed. Second, in our research, a notable 81% of enrolled patients presented with viral hepatitis, reflecting a considerable degree of homogenecity within the cohort. This diverse patient profile not only underscores the complexity of the cases but also positions itself as a valuable asset for future endeavors in comparing and analyzing the therapeutic effects of TACE. The broad spectrum of viral hepatitis cases captured in our study lays the foundation for comprehensive insights, serving as a robust reference point for nuanced evaluations and tailored interventions in TACE-based treatments. Thirdly, in this study, a meticulous analysis was conducted by stratifying tumor size into subcategories, specifically distinguishing between sizes less than 2cm and those ranging from 2 to 5 cm. This granular approach enhances the significance of our findings as it allows for a nuanced evaluation of the TACE treatment effects tailored to different tumor sizes. By segmenting the data in this manner, our research not only provides a comprehensive perspective on the diverse impact of TACE but also furnishes valuable insights for clinicians to reference and assess the efficacy of TACE in correlation with varying tumor sizes.

However, there are several limitations. First, the observation period was short (<3 years), which may not be sufficient to assess survival or recurrence rates. Second, this was a single-center study with a limited number of enrollees, especially with a scarcity of patients with decompensated cirrhosis. Third, as a retrospective study, the criteria for selecting patients who underwent TACE were subjective with multiple variables. Forth, there is a shortage of reference for an AFP level of 20 ng/mL for assessing prognosis.

Consequently, further extensive research with long-term follow-up is warranted to address these limitations and enhance our understanding of the usefulness of TACE for treating early-stage HCC.

## 5. Conclusions

The results of the current study suggest that TACE may be a treatment option for early-stage HCC when surgery or local therapy is not suitable. If patients are selected well, TACE can be considered even in advanced cirrhosis. Moreover, a CR to the first TACE procedure and a low AFP level were associated with a favorable prognosis in patients with early-stage HCC.

## Figures and Tables

**Table 1:** Patients’ baseline characteristics and response to the first TACE.

Variable	All Patients (*n* = 97)	Univariable Cox Proportional Hazard Model			
		HR	95% CI *p*-Value		*p* Value
Age, years	63.47 ± 11.02	0.98	0.94	1.03	0.598
Sex					
Male	69 (71.13%)	1.00			
Female	28 (28.87%)	1.44	0.58	4.01	0.4240
Underlying disease					
Hepatitis B + hepatitis C	79 (81.44%)	1.00			
Nonviral liver disease	18 (18.56%)	1.46	0.53	4.01	0.4540
Tumor size					
<2 cm	44 (43.36%)	1.00			
>2 cm but <5 cm	53 (54.64%)	1.72	0.69	4.26	0.2410
Presence of liver cirrhosis					
Yes	84 (86.60%)	85,450,000.00	0.00		0.9970
No	13 (13.40%)	1.00			
Child–Turcotte–Pugh class					
A	74 (76.20%)	1.00			
B + C	23 (23.71%)	2.29	0.95	5.53	0.0656
MELD	56 (57.73%)	1.18	1.07	1.30	0.0015
Serum AFP (ng/mL)					
<20	56 (57.73%)	1.00			
>20	41 (42.27%)	2.92	1.20	7.00	0.0178
PIVKA-II					
<40	54 (55.67%)	1.00			
>40	43 (44.33%)	1.48	0.62	3.49	0.3690
Serum total bilirubin (mg/dL)	1.00 ± 0.56	1.55	0.80	3.01	0.1950
Serum ALT (IU/L)	48.45 ± 65.05	0.99	0.98	1.01	0.5640
Serum sodium (mEq/L)	138.33 ± 2.70	0.90	0.77	1.06	0.2160
Secum creatinine (mg/dL)	0.92 ± 0.54	1.12	0.52	2.11	0.7570
Platelet count (×10^3^/mL)	122.3 ± 54.76	0.99	0.98	1.00	0.0669
Prothrombin time (INR)	1.21 ± 0.20	14.22	2.34	86.43	0.0039
Ascites					
Yes	9 (9.28%)	2.14	0.62	7.25	
No	88 (90.72%)	1.00			
Varices					
Yes	37 (38.14%)	2.00	0.85	4.73	0.1110
No	60 (61.86%)	1.00			
Initial response to TACE					
Complete	84 (86.60%)	1.00			
Incomplete	13 (13.40%)	2.26	0.75	6.78	0.1440

AFP, alpha-fetoprotein; ALT, alanine transaminase; CI, confidence interval; HR, hazard ratio; MELD, Model for End-stage Liver Disease; PIVKA-II, protein induced by vitamin K absence-II; TACE, transarterial chemoembolization.

## Data Availability

The data published in this study are available upon request from the first author.
